# Phomopsterone B Alleviates Liver Fibrosis through mTOR-Mediated Autophagy and Apoptosis Pathway

**DOI:** 10.3390/molecules29020417

**Published:** 2024-01-15

**Authors:** Mei-Lin Peng, Li-Jie Zhang, Yan Luo, Shi-Ying Xu, Xing-Mei Long, Jun-Li Ao, Shang-Gao Liao, Qin-Feng Zhu, Xun He, Guo-Bo Xu

**Affiliations:** 1 State Key Laboratory of Functions and Applications of Medicinal Plants & School of Pharmacy, Guizhou Medical University, Guian New District, Guiyang 550004, China; pengml@163.com (M.-L.P.); zhanglj319@163.com (L.-J.Z.); yanluo2688@163.com (Y.L.); xushiyinging@163.com (S.-Y.X.); dm214192@163.com (X.-M.L.); aojl90@163.com (J.-L.A.); lshangg@163.com (S.-G.L.); zhuqinfeng@gmc.edu.cn (Q.-F.Z.); 2 University Engineering Research Center for the Prevention and Treatment of Chronic Diseases by Authentic Medicinal Materials in Guizhou Province, Guian New District, Guiyang 550025, China; 3 Engineering Research Center for the Development and Application of Ethnic Medicine and TCM, Ministry of Education, Guiyang 550004, China

**Keywords:** phomopsterone B, liver fibrosis, autophagy, apoptosis, hepatic stellate cells

## Abstract

Liver fibrosis is the initial pathological process of many chronic liver diseases. Targeting hepatic stellate cell (HSC) activation is an available strategy for the therapy of liver fibrosis. We aimed to explore the anti-liver fibrosis activity and potential mechanism of phomopsterone B (PB) in human HSCs. The results showed that PB effectively attenuated the proliferation of TGF-β1-stimulated LX-2 cells in a concentration-dependent manner at doses of 1, 2, and 4 μM. Quantitative real-time PCR and Western blot assays displayed that PB significantly reduced the expression levels of α-SMA and collagen I/III. AO/EB and Hoechst33342 staining and flow cytometry assays exhibited that PB promoted the cells’ apoptosis. Meanwhile, PB diminished the number of autophagic vesicles and vacuolated structures, and the LC3B fluorescent spots indicated that PB could effectively inhibit the accretion of autophagosomes in LX-2 cells. Moreover, rapamycin and MHY1485 were utilized to further investigate the effect of mTOR in autophagy and apoptosis. The results demonstrated that PB regulated autophagy and apoptosis via the mTOR-dependent pathway in LX-2 cells. In summary, this is the first evidence that PB effectively alleviates liver fibrosis in TGF-β1-stimulated LX-2 cells, and PB may be a promising candidate for the prevention of liver fibrosis.

## 1. Introduction

Liver fibrosis, characterized by the massive accretion of extracellular matrix proteins (ECM) including collagens I/III, laminin, and alpha smooth muscle actin (α-SMA) in the liver, is associated with persistent hepatocellular damage due to viral infection (e.g., hepatitis B and C) and fatty liver [1,2]. Advanced liver fibrosis can evolve into liver dysfunction and trigger serious complications, eventually developing into cirrhosis and hepatocellular carcinoma. Hepatic stellate cells (HSCs) belong to resident nonparenchymal cells, bearing a crucial role in the development of liver fibrosis. In the case of liver injury, HSCs lose their cytoplasmic lipid droplets and are then activated, transdifferentiating into myofibroblasts, which ultimately result in ECM deposition [2,3,4]. Therefore, targeting the activation of HSCs is a prominent strategy for the therapy of liver fibrosis.

Apoptosis is a type of programmed cell death (PCD), featured as DNA fragmentation, chromatin condensation, membrane blebbing, and cell shrinkage [5]. In the process of apoptosis, the Bcl-2 gene family-encoded proteins and caspase, such as Bcl-2, Bax, and caspase 3/9, play central links and are key executors [6]. In recent years, ample studies have demonstrated that the promotion of HSC apoptosis is involved in anti-liver fibrosis [7,8]. Autophagy is another PCD that relies on autophagosomes to degrade unnecessary and damaged organelles and viral infection in cells [9,10]. It is orchestrated dynamically by several regulatory proteins in the formation of an autophagic membrane, the phagocytosis of autophagosomes, and the fusion with autophagosomes and lysosomes (e.g., LC3 and p62) [11,12]. To date, the participation of autophagy in the liver has been confirmed, although the role of autophagy in the pathogenesis of liver fibrosis is still controversial [13,14].

Natural products are promising sources for the discovery of novel active templates for the treatment of various liver diseases [15,16]. The unique 13(14→8) abeo-ergostane-type steroids (e.g., dankasterones A/B and periconiastone A) that possess wide bioactivities including antibacterial (against *Staphylococcus aureus* and *Enterococcus faecalis*), cytotoxicity (for murine P388 and MG-MID-5.41), and anti-inflammation have piqued the interest of chemists and pharmacologists in recent years [17,18,19,20,21]. Phomopsterone B (PB) is a novel C29 steroid with a 13(14→8) abeo-ergostane scaffold that was initially obtained from the natural extract of *Phomopsis* sp. TJ507A; it exhibited inhibitory activity against the NO production of RAW 264.7 induced by LPS [22]. In our efforts to discover anti-liver fibrosis natural products, PB was isolated from the secondary metabolites of *Aspergillus udagawae* collected from the Xingren coal area, as a promising cell proliferation inhibitory agent against LX-2 cells. In this paper, we report the anti-liver fibrosis effect of PB and its related mechanism on TGF-β1-stimulated LX-2 cells.

## 2. Results

### 2.1. PB Prevents the Proliferation, Activation, and Migration of LX-2 Cells

**Figure 1:**
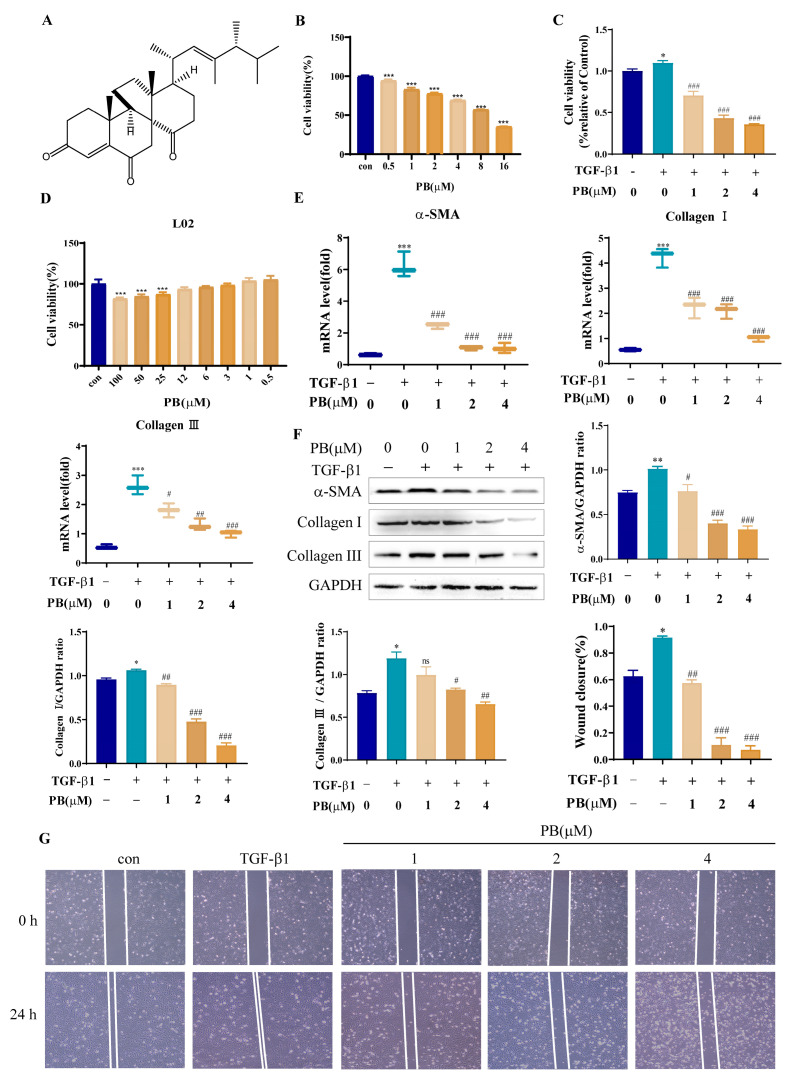
PB prevented the proliferation, activation, and migration of LX-2 cells. (**A**) The structure of phomopsterone B (PB); (**B**) Cell viability of LX-2 cells treated with PB; (**C**) PB dose-dependently inhibited the proliferation of TGF-β1 induced LX-2 cells. (**D**) Cell viability of L02 cells treated with PB; (**E**) PB suppressed the mRNA expression levels of α-SMA and Collagen I/III. (**F**) Western blot analysis of α-SMA and collagen I/III. (**G**) PB inhibited the migration of activated LX-2 cells observed via microscopic inspection. * *p* < 0.05, ** *p* < 0.01, *** *p* < 0.001 versus control; ^#^ *p* < 0.05, ^##^ *p* < 0.01, ^###^ *p* < 0.001 versus TGF-β1. ns: no significance.

LX-2 cells were firstly exposed to PB (the structure is shown in Figure 1A) at different concentrations for 24 h to explore the effects of PB on the proliferation of LX-2 cells. Then, an MTT assay was used to evaluate the viability of these cells. The outcomes indicated that PB effectively inhibited the proliferation of LX-2 cells induced with or without TGF-β1 in a concentration-dependent manner (Figure 1B,C). In addition, PB displayed no cytotoxicity to normal hepatocyte (L02) at concentrations below 12 μM (Figure 1D). Afterwards, we evaluated the effect of PB on HSC activation. With TGF-β1 stimulation of LX-2 cells, the expressions of hepatic fibrosis biomarker genes including α-SMA and collagen I/III were significantly increased in the qPCR experiments (Figure 1E), which showed suppressed expressions in the presence of PB. Meanwhile, the Western blot assay results also displayed that PB could significantly decrease the protein expression levels of α-SMA and collagen I/III in TGF-β1-stimulated LX-2 cells (Figure 1F). These results displayed that PB blocked TGF-β1-induced HSC activation, indicating PB possessed antifibrotic effects in the activated HSCs. In the cell wound healing assay, PB significantly decreased cell migration in comparison with the control and TGF-β1 groups (Figure 1G), suggesting that PB was able to inhibit the migration of LX-2 cells.

### 2.2. Molecular Docking

**Figure 2:**
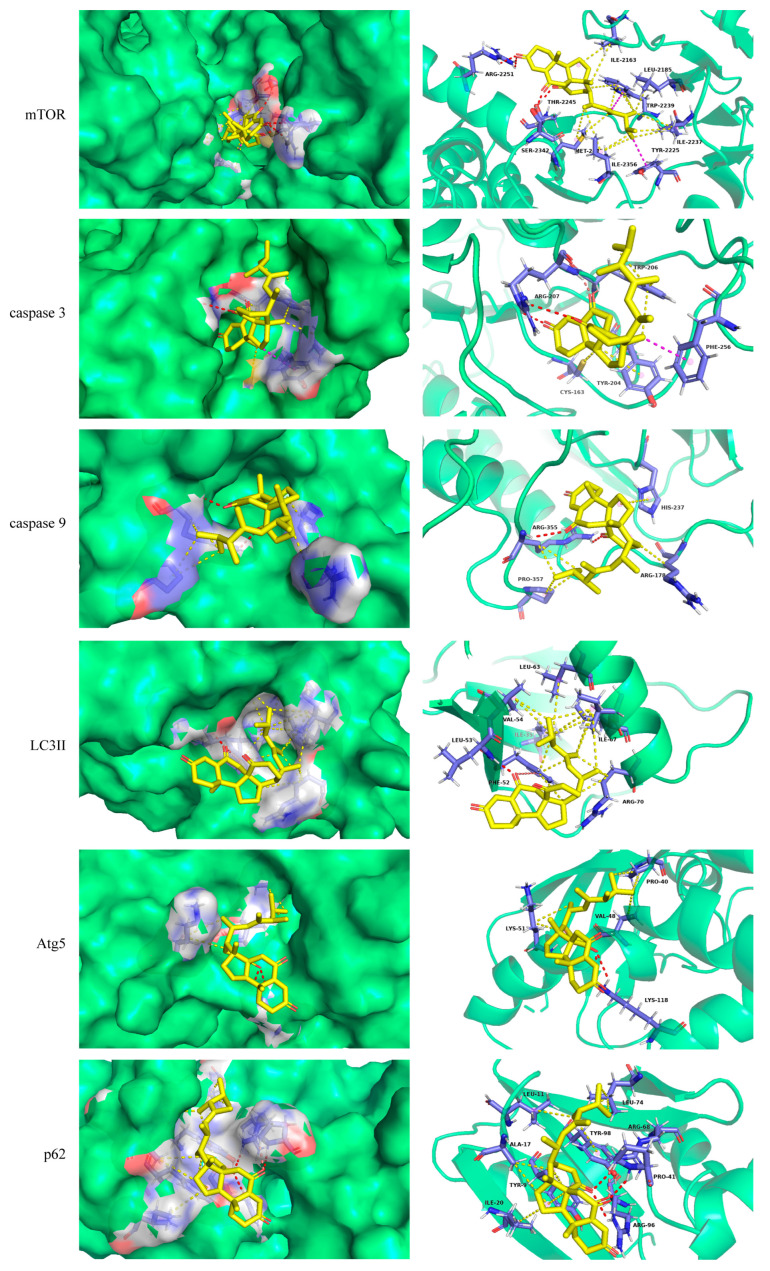
Binding modes of PB with proteins. Conventional hydrogen bond: Red dash line; Carbon hydrogen bond: Deepsalmon dash line; Alkyl, Pi-Alkyl: Yellow dash line; Pi-sigma: Magentas dash line; Protein: Limegreen; Amino acid: Slate; PB: Yellow.

Molecular docking is an efficient methodology used to explore the behavior of small molecules in the binding site of a target protein [23]. Considering the inhibitory effect of PB on LX-2 proliferation, molecular docking was employed to predict the binding affinity of PB to the possible targets in the apoptosis and autophagy signaling pathway, and six related proteins with crystal structures were selected to dock with PB. The results displayed that PB had considerable binding activity with mTOR, caspase-3, caspase-9, Atg5, LC3II, and p62 with binding energies of −9.4, −8.1, −7.3, −6.9, −6.9, and −7.8 kcal/mol, respectively. The molecular docking details of PB with each protein are shown in Figure 2, where PB binds to mTOR through several intermolecular interactions including hydrogen bonds (ARG:2251, SER:2342, and THR:2245). Similarly, the hydrogen bonds formed between PB and caspase-3, caspase-9, Atg5, LC3II, and p62 are listed in Table 1. These results suggest that PB may directly target mTOR, caspase-3, caspase-9, LC3II, Atg5, and p62 to disrupt their interactions, which subsequently affect the apoptotic and autophagic outcomes.

### 2.3. PB-Induced LX-2 Cell Apoptosis

**Figure 3:**
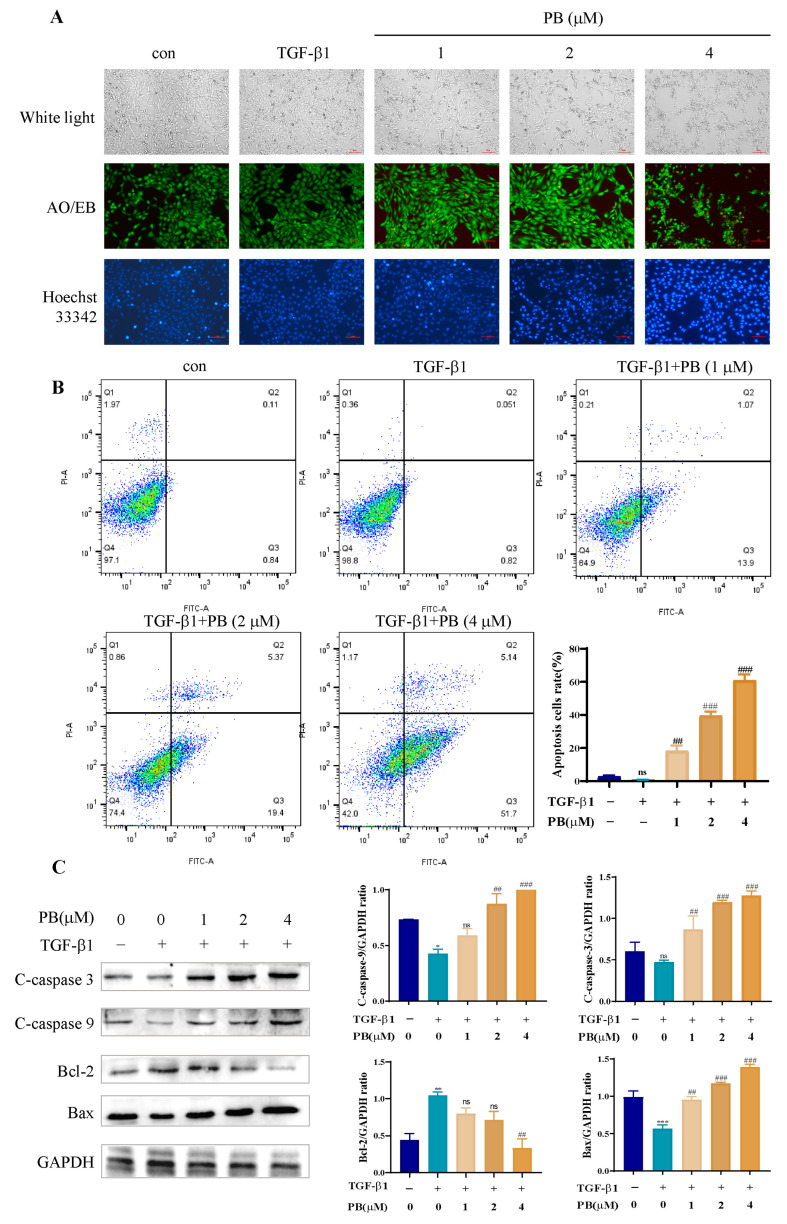
PB promoted apoptosis of LX-2 cells. (**A**) The apoptosis of LX-2 cell images demonstrated by Hoechest33342 and AO/EB staining. Scale bar: 10 μm. (**B**) Flow cytometry analysis of the apoptosis rate of LX-2 cells. (**C**) Western blotting analysis of the expression of the specific proteins. * *p* < 0.05, ** *p* < 0.01, *** *p* < 0.001 versus control; ^##^ *p* < 0.01, ^###^ *p* < 0.001 versus TGF-β1. ns: no significance.

The above results confirmed that PB could prohibit the proliferation of LX-2 cells and reduce the expression of fibrosis biomarkers. However, whether these effects are related to apoptosis was still not very clear. Therefore, the role of PB in the growth of LX-2 cells was investigated. After treatment of PB, cells exhibited cellular shrinkage (Figure 3A) and red fluorescence appeared, as visualized with AO/EB staining, which indicated that PB did induce cell apoptosis. Furthermore, Hoechst33342 staining also confirmed the conclusion, in which the apoptotic cells fluoresced bright blue, and the cell nuclei were obviously fragmented. The flow cytometry assay suggested that treatment with PB elevated the cell apoptotic ratio in comparison to the control (Figure 3B). Moreover, exposure to PB upregulated the protein expression of Bax and C-caspase-3/9 and decreased the expression level of Bcl-2 (Figure 3C). All results indicated that PB could significantly induce LX-2 cells apoptosis.

### 2.4. PB Suppressed Autophagy in Activated LX-2 Cells

**Figure 4:**
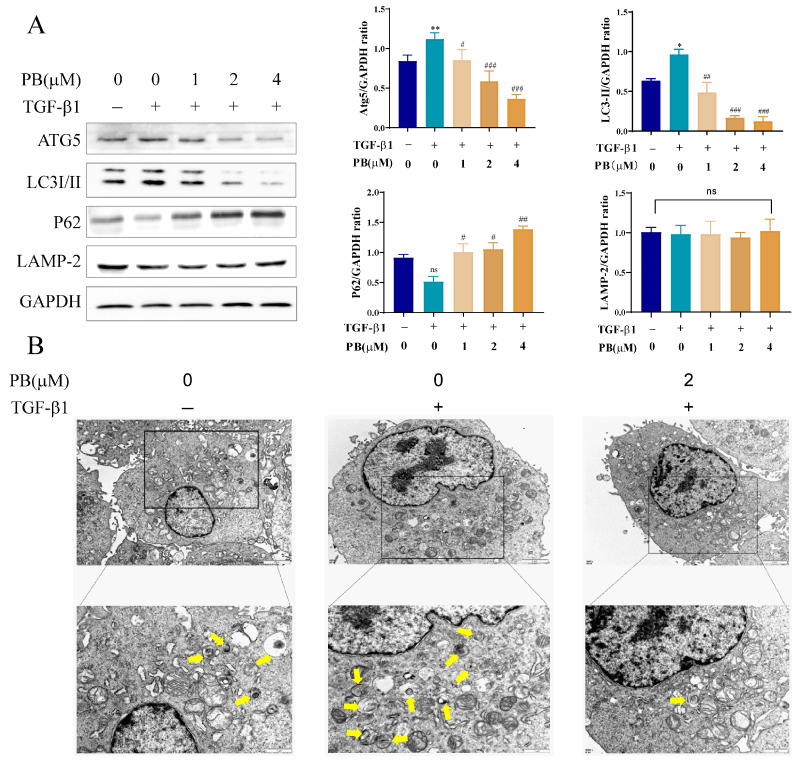
PB suppressed autophagy in LX-2 cells. (**A**) Western blot analysis of the expression of autophagy proteins (ATG5, LC3, P62, and LAMP-2) in LX-2 cells after being treated with PB for 24 h. (**B**) TEM analysis of the formation of autophagosomes in LX-2 cells after being treated with PB for 24 h. Yellow arrows indicate autophagosomes. Magnification times: 8000× (Scale bar: 2 μm, top) and 15,000× (Scale bar: 1 μm, bottom). * *p* < 0.05, ** *p* < 0.01 versus control; ^#^ *p* < 0.05, ^##^ *p* < 0.01, ^###^ *p* < 0.001 versus TGF-β1. ns: no significance.

**Figure 5:**
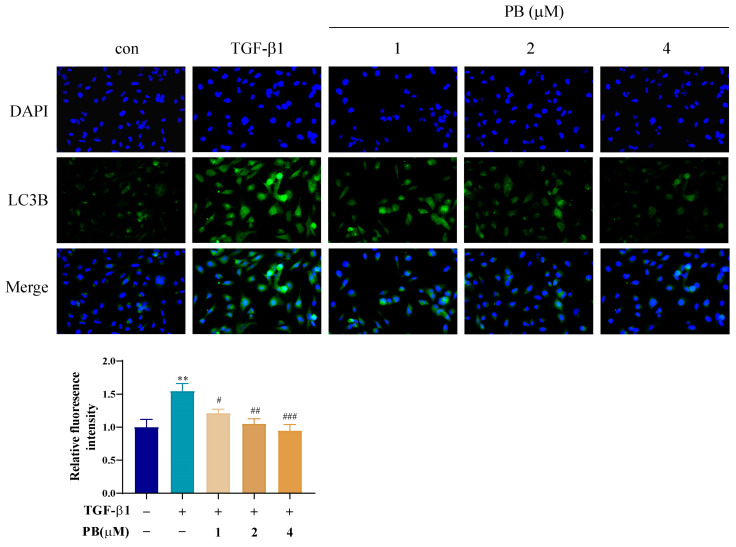
The immunofluorescence analysis of LC3-B in LX-2 cells treated with PB. ** *p* < 0.01 versus control; ^#^ *p* < 0.05, ^##^ *p* < 0.01, ^###^ *p* < 0.001 versus TGF-β1.

The effects of PB on the autophagy of activated LX-2 cells were further explored, focusing on the potential involvement of autophagy as an alternative cell death mechanism. Firstly, a Western blot assay was employed to determine the expressions of Atg5, LC3I/II, p62, and LAMP-2 (Figure 4A). Compared with the control group, the TGF-β1 group showed remarkably upregulated protein expressions of LC3II and Atg5 and a significantly diminished level of autophagosome transporter p62. Contrasted with TGF-β1, PB reversed the protein expressions of LC3II, Atg5, and p62 in the PB + TGF-β1 group. However, the LAMP-2 expression was practically unchanged with or without TGF-β1 and/or PF. Transmission electron microscopy (TEM) was also employed to observe the morphological changes in LX-2 cells. Compared to TGF-β1, PB reduced the populations of autophagic vesicles and vacuolated structures, which confirmed the ability of PB to diminish autophagosome accumulation (Figure 4B). To further prove that PB inhibits the formation of autophagosomes, we detected the accumulation of autophagosomes by using immunofluorescence staining, adopting the LC3B antibody and fluorescence microscopy to quantify fluorescent spots that correlated with the number of autophagosomes. The results showed that the number of LC3B fluorescent spots decreased in a dose-dependent manner (Figure 5). These data verified that PB could effectively inhibit the accretion of autophagosomes in LX-2 cells.

### 2.5. Inhibition of Autophagy Promotes Anti-Fibrosis Effects of PB In Vitro

**Figure 6:**
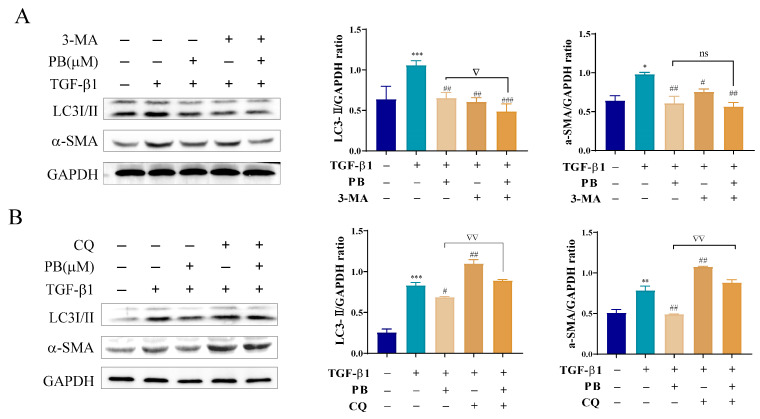
Western blot analysis of the expression of LC3II and α-SMA in treated LX-2 cells. (**A**) LX-2 cells treated with PB and/or 3-MA (5 mM). (**B**) LX-2 cells treated with PB and/or CQ (3 μM). * *p* < 0.05, ** *p* < 0.01, *** *p* < 0.01 versus control; ^#^ *p* < 0.05, ^##^ *p* < 0.01, ^###^ *p* < 0.001 versus TGF-β1. ^▽^ *p* < 0.05 and ^▽▽^ *p* < 0.01 versus 3-MA or CQ. ns: not significant.

**Figure 7:**
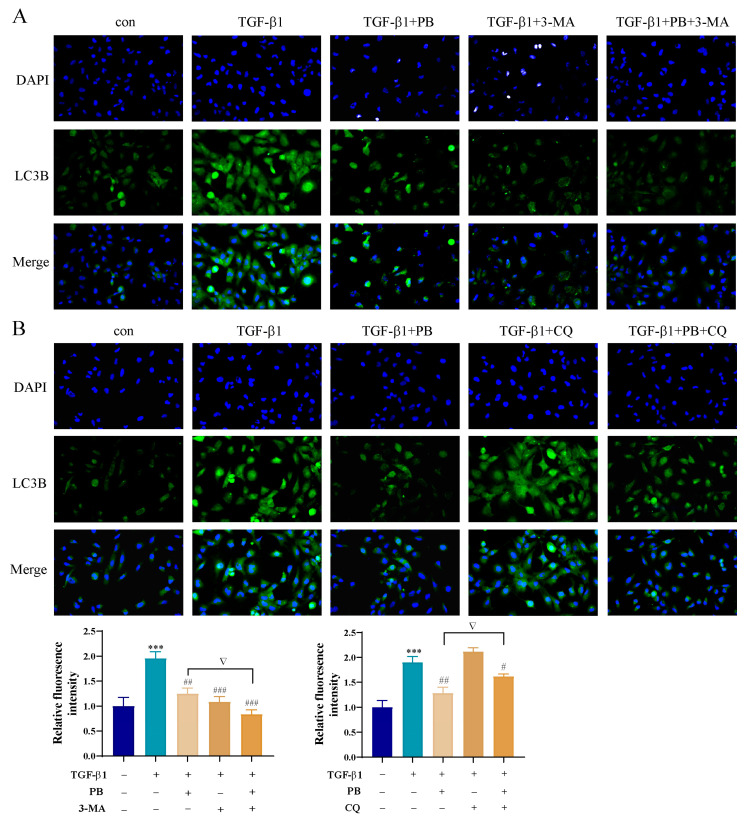
The immunofluorescence of LC3B in LX-2 cells treated with (**A**) PB, 3-MA, and PB+3-MA; and (**B**) PB, CQ, and PB+CQ. *** *p* < 0.01 versus control; ^#^ *p* < 0.05, ^##^ *p* < 0.01, ^###^ *p* < 0.001 versus TGF-β1. ^▽^ *p* < 0.05 versus 3-MA or CQ.

The expression of related proteins in autophagosomes (e.g., LC3II) is an important index reflecting the process of autophagy. 3-Methyladenine (3-MA) is generally used as an autophagy inhibitor to interfere with the initial formation of autophagosome. Chloroquine (CQ) is a binding inhibitor of autophagosomes and lysosomes. To investigate the influence of PB on autophagy at different stages in liver fibrosis, LX-2 cells were treated with TGF-β1, 3-MA, and PB. The results showed that the expressions of LC3II and liver fibrosis marker α-SMA were markedly downregulated in contrast with those of the TGF-β1 group according to Western blot analysis. These results demonstrated that PB was able to reduce liver fibrosis by inhibiting autophagosome formation (Figure 6A). Meanwhile, the accumulation of LC3II and the upregulation of the expression of α-SMA were observed after treatment with CQ in LX-2 cells, while the level of these indicators was markedly decreased with treatment with PB and “CQ + PB” (Figure 6B), implying that the elevation of LC3II was not due to the blockade of autophagosome–lysosome fusion by PB. The immunofluorescence experiments directly illustrated the same results (Figure 7), indicating that PB inhibited the formation of autophagosomes. Collectively, these results implied that PB suppressed the activation of LX-2 cells by attenuating autophagy.

### 2.6. PB Regulates Autophagy and Apoptosis through mTOR-Dependent Mechanism in LX-2 Cells

**Figure 8:**
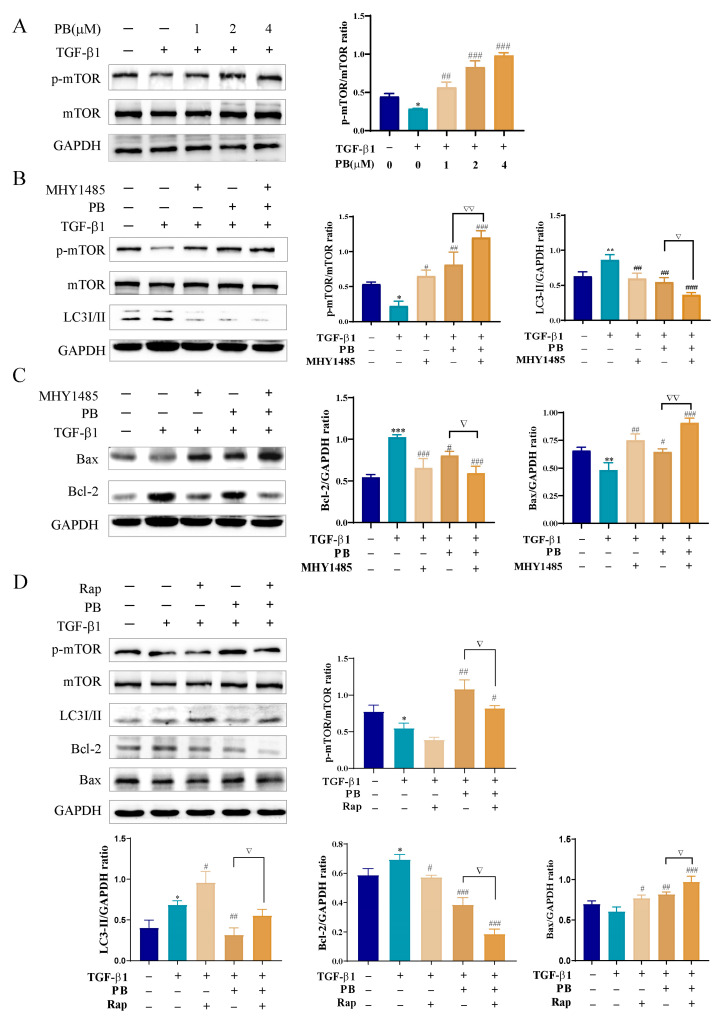
PB regulated autophagy and apoptosis of LX-2 cells via mTOR-dependent mechanism. (**A**) Western blot analysis of p-mTOR/mTOR levels after PB treatment for 24 h. (**B**–**D**) The protein expression levels of p-mTOR/mTOR, LC3I/II, Bax, and Bcl-2 in LX-2 cells treated with or without 2 μM PB, 10 µM MHY1485, and 100 μM rapamycin for 24 h. * *p* < 0.05, ** *p* < 0.01, *** *p* < 0.001 versus control; ^#^ *p* < 0.05, ^##^ *p* < 0.01, ^###^ *p* < 0.001 versus TGF-β1; ^▽^ *p* < 0.05, ^▽▽^ *p* < 0.01 versus MHY1485 or Rap; ns: no significance.

Mammalian target of rapamycin (mTOR) is a highly conserved eukaryotic serine/threonine protein kinase that is involved in cell growth, metabolism, proliferation, and autophagy [24]. We thus investigated whether PB mediated apoptosis and autophagy through mTOR-dependent signaling pathways. As shown in Figure 8A, the p-mTOR/mTOR ratio was dramatically increased after being treated with PB. Then, rapamycin (Rap, mTOR inhibitor) and MHY1485 (mTOR activator) were utilized to further explore the role of mTOR in autophagy and apoptosis. After the treatment of LX-2 cells with PB or MHY1485, the levels of p-mTOR/mTOR and apoptosis biomarker Bax were significantly elevated, whereas the levels of LC3II/I and Bcl-2 were reduced in contrast with those in the TGF-β1 group (Figure 8B,C). As shown in Figure 8D, the levels of p-mTOR/mTOR, Bcl-2, and LC3II remarkably decreased and the level of Bax was obviously upregulated by the treatment with PB and Rap. The aforementioned results verified that PB regulated autophagy and apoptosis via the mTOR-dependent pathway.

## 3. Discussion

Liver fibrosis is a wound-healing response to tissue injury featuring an abundant accumulation of ECM [25]. Currently, most biological or chemical agents under investigation focus on obviating the etiology, relieving inflammation, blocking the activation of HSCs, and promoting the apoptosis of activated HSCs [25,26]. In this study, PB could effectively prohibit the proliferation, migration, apoptosis, and activation of LX-2 cells by reducing the expressions of α-SMA and collagen I/III.

Apoptosis is a programmed cell death process that is critical for tissue growth, development, and homeostasis in metazoans [27]. The induction of apoptotic cell death is commonly assessed using markers such as apoptotic inhibitory factor Bcl-2 and apoptotic factor Bax. A part of the family of cysteine proteases, caspases play a vital role in cell apoptosis as central regulators [28]. In this study, we found that treatment of LX-2 cells with PB obviously upregulated the expression levels of cleaved caspase-3/9 and Bax, and inhibited the level of Bcl-2, suggesting that PB could markedly induce cell apoptosis.

Autophagy degrades and recycles organelles and macromolecules for cells to sustain internal environmental homeostasis. However, the excessive activation of autophagy leads to suicidal cell death [29]. Autophagy is closely mediated by autophagy-related genes (e.g., Atg5, LC3II) and proteins. In the formation of autophagosomes, autophagic vacuoles are firstly constructed in cells when stimulated by the external environment. Then, Atg5 complexes are quickly created and combined with autophagic vacuoles. The soluble form (LC3I) is transformed into a lipid-soluble form (LC3II), which further couples with autophagic vacuoles to form autophagosomes, and, finally, autophagosomes fuse with lysosomes, followed by decomposing the inner membrane and its contents [29,30]. In the current study, the expression levels of Atg5, LC3II, and α-SMA were obviously suppressed in the activated LX-2 cells after treatment with 3-MA or/and PB, suggesting PB could effectively inhibit LX-2 cell activation via autophagy. In the process of autophagy, p62 (SQSTM1) is usually located on the autophagosome in the form of a complex with the LC3II protein, ubiquitinating degraded contents in the autophagosomes [30]. As an autophagosome degrades, p62 degrades as well; thus, the p62 level is correlated with the emergence and development of autophagy [31]. In this study, the increasing level of p62 indicated that PB blocked the synthesis of autophagic vesicles instead of improving the autophagic degradation in LX-2 cells. LAMP-2 is situated on the lysosomal membrane to ensure its integrity [32]. The fact that there was no significant change in LAMP-2 further indicated that PB acted at the prephage formation stage rather than the degradation stage.

Ample evidence suggests that mTOR is involved in regulatory signals concerning intracellular growth factors, energy levels, and nutritional status through the PI3K/Akt pathways. Lee et al. reported that tenofovir disoproxil fumarate promoted the apoptosis of activated HSCs by downregulating the PI3K/Akt/mTOR signaling pathway to ameliorate liver fibrosis [33]. Wang et al. reviewed the mTOR-mediated autophagy in liver diseases related to AMPK/mTOR, Ras/Raf/MEK/ERK/mTOR, PI3K/AKT/mTOR, and their cross-talk signaling pathways [34]. When mTOR is activated, a series of cascade effects are activated to affect apoptosis and autophagy [35]. In this study, PB significantly raised the p-mTOR level in the LX-2 cells after treatment with PB. mTOR inhibitor (Rap) and activator (MHY1485) were further employed to investigate whether PB mediated autophagy and apoptosis via mTOR. The results showed that PB increased p-mTOR and Bax and reduced LC3 II and Bcl-2, verifying that PB regulated autophagy and apoptosis via the mTOR-dependent pathway. Additionally, the mechanism of liver fibrosis is extremely complicated, and multiple signaling pathways are involved. Meanwhile, PB belongs to 13(14→8) abeo-ergostane-type steroids, and whether it could act on steroid receptors to affect the downstream signaling pathway in LX-2 cells for its anti-liver fibrosis effect needs to be further studied.

## 4. Materials and Methods

### 4.1. Materials

*Aspergillus udagawae* (collected from the Xingren Coal area) was cultured on a rice medium (200 g/flask × 30) in an incubator at 28 °C for 25 days. A crude extract was acquired from the fermentation substance and soaked with EtOH (35 L × 2) at room temperature (75.0 g). The crude extract was subject to a silica gel column (Φ 60 mm × 500 mm) and eluted with a gradient solvent system of petroleum ether-acetone (10:1 → 0:1) to produce four fractions (Fr. A–D), according to TLC analysis. Then, Fr. B was parted with gradient elution with petroleum ether-ethyl acetate (10:1 → 1:1) to yield Frs. B1–B4. B1 was further isolated using Sephadex LH20 with chloroform–methanol (3:2, *V*/*V*) to provide subfractions B1A–B1C. Finally, phomopsterone B (PB, 1.7 mg, t_R_ = 20 min) was obtained via semipreparative HPLC eluting with MeOH-H_2_O (87:13, *V*:*V*) solution with 0.1% formic acid. The purity of PB was over 95%. ESI MS *m/z* 439.3 [M + H]^+^; ^1^H-NMR (600 MHz, CDCl_3_): *δ* 6.37 (1H, s, H-4), 5.04 (1H, d, *J* = 9.0 Hz, H-22), 2.81 (1H, t like, H-9), 2.44–2.66 (6H, overlapped, H-2/7/15/20), 2.09–1.97 (4H, overlapped, H-1/11/16), 1.66–1.80 (4H, overlapped, H-11/12/16/24), 1.51 (3H, s, H-29), 1.50–1.40 (2H, overlapped, H-17/25), 1.26 (3H, s, H-19), 1.00 (3H, d, *J* = 6.6 Hz, H-21), 0.91 (3H, s, H-18), 0.91 (3H, overlapped, H-28), 0.84 (3H, d, *J* = 6.6 Hz, H-27), 0.75 (3H, d, *J* = 6.6 Hz, H-26); ^13^C-NMR (150 MHz, CDCl_3_): δ 215.0 (C-14), 200.1 (C-6), 199.3 (C-3), 156.0 (C-5), 137.6 (C-23), 128.1 (C-22), 126.6 (C-4), 62.2 (C-8), 53.5 (C-13), 50.7 (C-24), 50.4 (C-17), 49.2 (C-9), 41.2 (C-7), 39.0 (C-12), 38.9 (C-1), 38.1 (C-15), 36.0 (C-10), 34.5 (C-2), 32.2 (C-20), 31.0 (C-25), 25.1 (C-11), 24.1 (C-19), 22.7 (C-21), 22.5 (C-16), 21.8 (C-26), 20.4 (C-27), 16.8 (C-18), 16.7 (C-28), 12.7 (C-29). The structure of PB was established by contrasting NMR and MS data with those reported [22].

The antibodies including collagen I/III, α-SMA, Bax, cleaved caspase-3/9, p62, LC3, ATG5, and GAPDH were obtained from Proteintech Co., Ltd. (Wuhan, China). Bcl-2 was provided by Boaosen Co., Ltd. (Beijing, China). Fetal bovine serum (FBS) was procured from HyClone LLC (Logan, UT, USA). Dulbecco’s modified Eagle medium (DMEM) was supplied by Gibco LLC (New York, NY, USA). Penicillin and streptomycin were purchased from New Cell & Molecular Co., Ltd. (Suzhou, China). 3-Methyladenine and chloroquine were acquired from MedChemExpress LLC (Princeton, NJ, USA).

### 4.2. Cell Culture and Viability Assay

LX-2 cells were supplied by Shanghai Zhong Qiao Xin Zhou Biotechnology Co., Ltd. (Shanghai, China). Cell lines were cultured in DMEM, replenished with 10% (*V*/*V*) FBS and 100 U/mL penicillin/streptomycin, and grown in an incubator at 37 °C with 5% CO_2_. LX-2 cells were counted after detachment with trypsin. A total of 1 × 10^4^ cells per well with or without 10 ng/mL TGF-β1 and/or PB (1, 2, or 4 μM) were added into a 96-well plate and cocultured for 24 h. After, 5 mg/mL of MTT was added to the wells, which were cultured for another 4 h. After removal of the suspension, the formazans were dissolved in DMSO and detected using a multifunction microplate reader (Biotek, Bad Friedrichshall, Germany) at a wavelength of 490 nm.

### 4.3. Cell Wound Healing Assay

LX-2 cells with a density of 1 × 10^5^ were seeded in 6-well plates and incubated for 24 h. When the cell monolayers reached 80% confluency, a straight line was gently drawn with a sterile pipette tip. The monolayer membrane was washed using PBS three times, and then specified concentrations (1, 2, and 4 μM) of a compound containing 1% fetal bovine serum was added into each well. The images of cells were photographed with a SOPTOP ICX41 (SunGrant, Beijing, China) at 0 and 24 h. Each experiment was conducted in triplicate.

### 4.4. Cell Fluorescence Assay

A total of 1 × 10^5^ LX-2 cells were placed into six-well plates. The activated LX-2 cells were incubated with treatment with PB (1, 2, and 4 μM) for 24 h. The cells were dyed with acridine orange (AO)/ethidium bromide (EB) (Yuanye Biotech, Shanghai, China) in a dark incubator for 5 min, while the time for dying with Hoechst 33342 (Solarbio, Beijing, China) staining was 30 min. Finally, the cells’ status was determined using a fluorescence microscope (Olympus, Shinjuku City, Japan).

### 4.5. Immunofluorescent Staining

We employed 4% paraformaldehyde employed to fix the LX-2 cells for 30 min, which we then diosmosed with 1% Triton X-100 (Solarbio) and closed with 5% BSA (Solarbio) for 1 h at room temperature. The cells and anti-LC3B antibody were cocultured overnight at 4 °C. After being washed with PBS, cells were incubated with fluorescein (FITC)-conjugated affinipure donkey antirabbit lgG(H+L) for 1 h and counterstained with DAPI for another 5 min for observation of nuclei. Images were obtained with an inverted fluorescence microscope (Mshot, Guangzhou, China). Fluorescence staining was quantified usign Image J software (imagej.nih.gov/ij/).

### 4.6. Flow Cytometric Analysis

The apoptotic cell assay was analyzed with an Annexin FITC/PI double-staining procedure. In short, LX-2 cells and PB with a specific dose were cocultured for 24 h. After being digested into single cells with EDTA-free trypsin, the cells were then stained for 10–15 min, in line with the instructions for the Annexin FITC/PI Apoptosis Detection Kit (Jiangsu KeyGen Biotech Co., Ltd., Nanjing, China). Finally, flow cytometry was used to analyze the stained cells.

### 4.7. Quantitative Real-Time PCR

Total RNA of LX-2 cells was acquired by using Trizol reagent (Ambion, Austin, TX, USA) complying with its instructions. cDNA synthesis was performed on HiScript^®^ II Q Select RT SuperMix for qPCR (Vazyme, Nanjing, China) in triplicate. The level of GAPDH (human) RNA was applied to standardize the data. PCR primer sequences are listed in Table 2.

### 4.8. Western Blot Analysis

LX-2 cells were lysed for the extraction of total proteins. A BCA assay kit (Beijing, China, Solarbio) was employed to detect the protein concentration. An equal amount of proteins was mounted onto 10% SDS-PAGE and transferred onto PVDF membranes (Sigma-Aldrich, St. Louis, MO, USA). After blocking with 5% fat-free milk at room temperature for 1 h, the membranes were incubated overnight at 4 °C with primary antibodies including α-SMA, collagen I/III, cleaved-caspase-3/9, Bax, Bcl-2, p62, LC3, ATG5, and GAPDH. Subsequently, the membranes were treated with secondary antibodies at room temperature for 2 h. All protein bands were captured and analyzed with a ChemiDoc™ imaging system (Hercules, Wilmington, DE, USA).

### 4.9. Molecular Docking

Molecular docking is an efficient methodology used to predict the binding interaction of a ligand molecule with a target protein [36]. The 3D structures of proteins LC3II (PDB ID: 5GMV), Atg5 (PDB ID: 4TQ1), caspase-3 (PDB ID: 5I9B), caspase-9 (PDB ID: 2AR9), p62/SQSTM1 (PDB ID: 6JM4), and mTOR (PDB ID: 4JSV) were obtained from the PDB database (https://www.rcsb.org/, accessed on 2 September 2023). The 2D structure of PB was drawn using ChemBio 2D Ultra 14.0 and transformed into a PDB file format using PyMOL version 2.5.2. Energy minimizations of ligands were executed using ChemBio 3D Ultra 14.0 using standard protocols. Molecular docking studies were completed with AutoTock Vina version 1.1.2. The interactions between PB and caspase-3, caspase-9, SQSTM1, mTOR, Atg5, and LC3II were analyzed using Discovery Studio 2016 Client and PyMOL [37].

### 4.10. Statistical Analysis

Data were dealt with using GraphPad Prism 8.0 software using one-way ANOVA and *t*-test to evaluate significant differences between multiple and two groups, respectively. All experiments were repeated three times. Values are expressed as mean ± SD. Student’s *t*-test was employed to compare the control and model groups (for two groups). *p*-value < 0.05 was considered as statistically significant. * *p* < 0.05, ** *p* < 0.01, *** *p* < 0.001 versus control; ^#^ *p* < 0.05, ^##^ *p* < 0.01, ^###^ *p* < 0.001 versus TGF-β1. ns: no significance.

## 5. Conclusions

In conclusion, this is the first evidence that PB can effectively alleviate liver fibrosis in TGF-β1-stimulated LX-2 cells by suppressing autophagy and promoting apoptosis via the mTOR-dependent pathway. These results suggest that PB is a promising candidate for the prevention and treatment of liver fibrosis.

## Figures and Tables

**Table 1:** Molecular docking of PB with possible proteins.

Protein	Binding Energy kcal/mol	Hydrogen Bond Interaction	Distance Å
mTOR	−9.4	ARG-2251	2.2, 2.7
		SER-2342	2.0
		THR-2245	2.0
caspase 3	−8.1	ARG-207	1.9, 2.4, 3.4
		TRP206	2.8
caspase 9	−7.3	ARG-355	2.3, 3.0
Atg5	−6.9	LYS118	2.8, 3.1
LC3II	−6.9	LEU-53	1.8
		PHE-52	2.9
p62	−7.8	PRO41	2.5
		ARG68	2.8
		ARG96	3.0

**Table 2:** The primer sequences used for real-time qPCR.

**Gene**	**Forward**	**Reverse**
α-SMA	TCATGGTCGGTATGGGTCAG	CGTTGTAGAAGGTGTGGTGC
Collagen I	TGGAGAGGAAGGAAAGCGAG	ACCAGCTTCACCAGGAGATC
Collagen III	AAAAGGGGAGCTGGCTACTT	GAATTTCTGGGTTGGGGCAG
GAPDH	TCAAGAAGGTGGTGAAGCAGG	TCAAAGGTGGAGGAGTGGGT

## Data Availability

The data are contained within the article. Additional information is available on request from the corresponding author.
